# Altered 5-HT_2C _receptor agonist-induced responses and 5-HT_2C _receptor RNA editing in the amygdala of serotonin transporter knockout mice

**DOI:** 10.1186/1471-2210-11-3

**Published:** 2011-04-07

**Authors:** Pablo R Moya, Meredith A Fox, Catherine L Jensen, Justin L Laporte, Helen T French, Jens R Wendland, Dennis L Murphy

**Affiliations:** 1 Laboratory of Clinical Science, National Institute of Mental Health, National Institutes of Health, Bethesda MD, USA

**Keywords:** 5-HT_2C_, RNA editing, SERT, anxiety, amygdala

## Abstract

**Background:**

The serotonin 5-HT_2C _receptor (5-HT_2C_R) is expressed in amygdala, a region involved in anxiety and fear responses and implicated in the pathogenesis of several psychiatric disorders such as acute anxiety and post traumatic stress disorder. In humans and in rodent models, there is evidence of both anxiogenic and anxiolytic actions of 5-HT_2C _ligands. In this study, we determined the responsiveness of 5-HT_2C_R in serotonin transporter (SERT) knockout (-/-) mice, a model characterized by increased anxiety-like and stress-responsive behaviors.

**Results:**

In the three-chamber social interaction test, the 5-HT_2B/2C _agonist mCPP decreased sociability and sniffing in SERT wildtype (+/+) mice, both indicative of the well-documented anxiogenic effect of mCPP. This 5-HT_2C_-mediated response was absent in SERT -/- mice. Likewise, in the open field test, the selective 5-HT_2C _agonist RO 60-0175 induced an anxiogenic response in SERT +/+ mice, but not in SERT -/- mice. Since 5-HT_2C_R pre-mRNA is adenosine-to-inosine (A-to-I) edited, we also evaluated the 5-HT_2C_R RNA editing profiles of SERT +/+ and SERT -/- mice in amygdala. Compared to SERT +/+ mice, SERT-/- mice showed a decrease in less edited, highly functional 5-HT_2C _isoforms, and an increase in more edited isoforms with reduced signaling efficiency.

**Conclusions:**

These results indicate that the 5-HT_2C_R in the amygdala of SERT -/- mice has increased RNA editing, which could explain, at least in part, the decreased behavioral responses to 5-HT_2C _agonists in SERT -/- mice. These alterations in 5-HT_2C_R in amygdala may be relevant to humans with SERT polymorphisms that alter SERT expression, function, and emotional behaviors.

## Background

The serotonergic system has been implicated in the pathophysiology and treatment of mood and anxiety disorders, as well as schizophrenia [1,2]. The neurotransmitter serotonin (5-hydroxytryptamine, 5-HT) influences neuronal activity via 14 5-HT receptors termed 5-HT_1 _through 5-HT_7 _(for a review, see [3]). The 5-HT_2C _receptor (5-HT_2C_R) has been implicated in normal and altered function of neural circuitries involved in these neuropsychiatric disorders via genetic, immunohistochemical and pharmacological approaches [2,4,5]. The 5-HT_2C_R is a G-protein coupled receptor (GPCR) coupled to PLC and PLA_2_, although additional signaling cascades have also been described [6-8].

Our previous work indicates that 5-HT transporter (SERT) knockout (-/-) mice are a valid model to study anxiety-related behaviors. These mice exhibit a complex phenotype dominated by anxiety, exaggerated stress responsiveness, and other physiological effects such as obesity and type 2 diabetes-like symptoms, all of which have been previously associated with 5-HT_2C_R genetic deficiencies ([9-12]; for a full review of SERT -/- mice, see [1]). Qu and colleagues [13] found a reduction in 5-HT_2_R-induced arachidonic acid release in multiple brain regions including the basolateral amygdaloid complex of SERT -/- mice [13,14]. Further, we previously showed increased 5-HT_2C_R binding sites with no mRNA changes in the amygdala of SERT -/- mice compared SERT +/+ mice [15]. The exact mechanisms responsible for the anxiety-like phenotype of SERT-/- mice are, however, not completely understood.

In humans and rodents, 5-HT_2C_R pre-mRNA is subject to adenosine-to-inosine (A-to-I) RNA editing [16,17]. These base changes may result in an amino acid/protein different from those encoded by genomic DNA. It has been shown that RNA editing alters the G-protein efficiency of the 5-HT_2C_R and its intracellular downstream effects and interactions with both endogenous and exogenous receptor agonists, as well as desensitization mechanisms and constitutive activity [17-20]. It is noteworthy that the 5-HT_2C_R is the only example among the hundreds of GPCRs which exhibits this post-transcriptional processing [5].

A body of evidence suggests there are alterations in the 5-HT_2C_R editing pattern in patients with certain neuropsychiatric diseases, and it has been suggested that 5-HT_2C_R RNA editing may play a role in anxiety and depression [21-25]. The aim of the current study was to evaluate the status of 5-HT_2C_R-mediated anxiety-like behaviors in SERT -/- mice. The finding that SERT -/- mice were unresponsive to systemic administration of 5-HT_2C_R agonists at doses that elicited anxiogenic responses in SERT +/+ mice prompted us to further investigate the RNA editing profile of the 5-HT_2C_R in SERT -/- mice. We chose the amygdala as our primary target, since this region is critical in anxiety-related behaviors in rodents, non-human primates and humans [2,26-28].

## Results

### Behavioral analysis

#### Social interaction test

**Figure 1:**
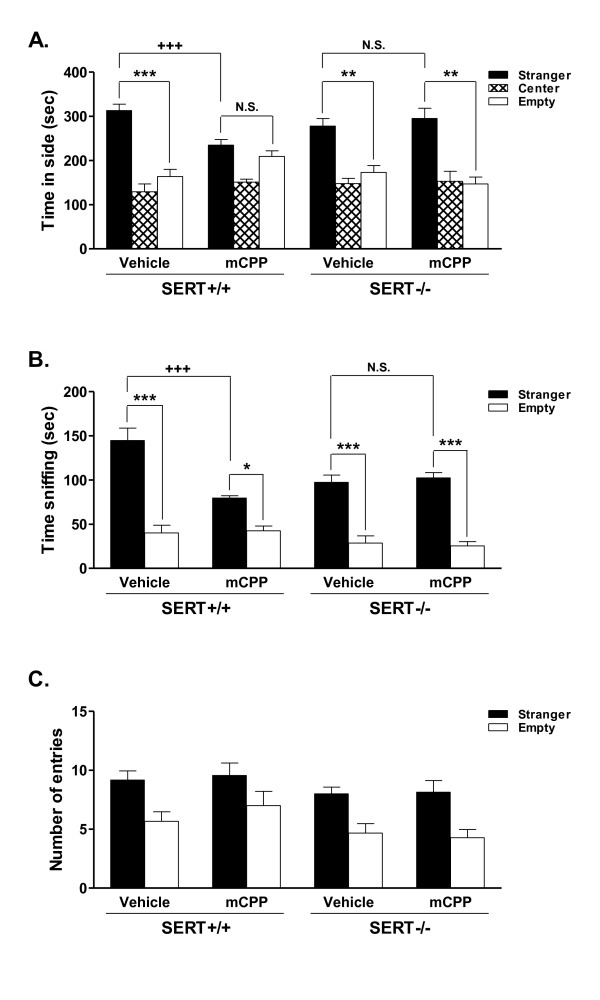
**Effects of mCPP in the social interaction test in SERT +/+ and -/- mice**. **1A**. Effects of mCPP on "sociability". SERT +/+ and -/- mice were given vehicle or mCPP 1 mg/kg ip 30 min prior to testing. mCPP decreased sociability (indicating increased anxiety) in SERT +/+ mice, with no effects in SERT -/- mice. **1B.** Effects of mCPP on "sniffing." SERT +/+ and SERT -/- mice were given vehicle or mCPP 1 mg/kg ip 30 min prior to testing. mCPP decreased sniffing in SERT +/+ mice (indicating increased anxiety), with no effects in SERT -/- mice. **1C.** Effects of mCPP on locomotor activity. There was no effect of mCPP 1 mg/kg ip administered 30 min prior to testing on the number of entries into the different chambers. Data represent the mean ± SEM, 7 animals per group. *** p < 0.001, ** p < 0.01, and * p < 0.05 vs. the stranger chamber in mice of the same genotype in the same drug condition; +++ p < 0.01 vs. mice of the same genotype given vehicle; N.S. not significant.

In the social interaction test we first assessed "sociability" (the preference for spending time in the stranger side vs. the empty side) [main effects of side (F_1,22 _= 61.53, p < 0.0001); genotype (F_1,22 _= 0.92, N.S.) and drug (F_1,22 _= 1.72, N.S.); side × genotype interaction (F_1,22 _= 1.10, N.S.), side × drug interaction (F_1,22 _= 1.18, N.S.), genotype × drug interaction (F_1,22 _= 0.56, N.S.) and side × drug × genotype interaction (F_1,22 _= 6.87, p = 0.016)]. In vehicle-treated mice, there were no significant differences in sociability between SERT +/+ and -/- mice (Figure 1A). In SERT +/+ mice, administration of mCPP reduced sociability, indicative of its anxiogenic effect, whereas mCPP had no effect in SERT -/- mice, reflecting a diminished responsiveness of the 5-HT_2C_R (Figure 1A).

We next assessed "sniffing" (time spent sniffing the stranger cage vs. the empty cage) [main effects of side (F_1,22 _= 183.26, p < 0.0001), genotype (F_1,22 _= 5.59, p = 0.027) and drug (F_1,22 _= 8.97, p = 0.007); side × genotype interaction (F_1,22 _= 0.002, N.S.), side × drug interaction (F_1,22 _= 6.98, p = 0.015), genotype × drug interaction (F_1,22 _= 10.13, p = 0.004), and side × drug × genotype interaction (F_1,22 _= 11.61, p = 0.003)]. Vehicle-treated SERT +/+ and -/- mice both spent more time sniffing the stranger vs. the empty cage (Figure 1B). However, mCPP-treated SERT +/+ mice spent significantly less time sniffing the stranger cage compared to vehicle-treated SERT +/+ mice, whereas mCPP was without such an effect in SERT -/- mice (Figure 1B).

To rule out a possible role for changes in locomotor activity, we also assessed the number of entries to each side chamber [main effects of side (F_1,22 _= 28.23, p < 0.0001), genotype (F_1,22 _= 5.69, p = 0.026) and drug (F_1,22 _= 0.56, N.S.); side × genotype interaction (F_1,22 _= 0.20, N.S.), side × drug interaction (F_1,22 _= 0.03, N.S.), genotype × drug interaction (F_1,22 _= 0.56, N.S.), and side × drug × genotype interaction (F_1,22 _= 0.33, N.S.)]. There were no significant differences in the number of entries to the side chambers based on genotype or drug administration, suggesting that differences in locomotor activity did not play a role in the differences in anxiogenic responses elicited by mCPP in SERT +/+ mice (Figure 1C).

**Figure 2:**
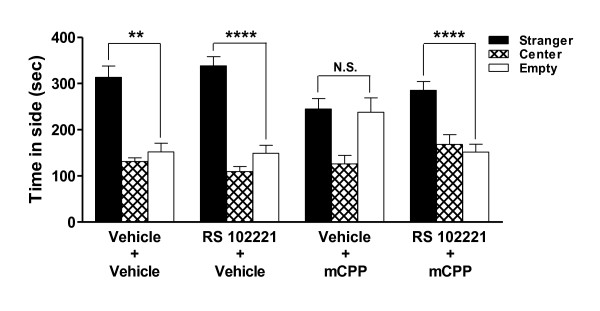
**Effects of pretreatment with the selective 5-HT_2C _antagonist RS 102221 on the anxiogenic effects of mCPP on "sociability" in the social interaction test in wildtype C57BL/6J mice**. Mice were given vehicle or RS 102221 1 mg/kg ip 15 min prior to vehicle or mCPP 1 mg/kg ip. RS 102221 antagonized the anxiogenic effects of mCPP on "sociability." Data represent the mean ± SEM, 5-7 animals per group. **** p < 0.0001 and ** p < 0.01 vs. the stranger chamber in mice of the same genotype in the same drug condition; N.S. not significant.

Administration of the selective 5-HT_2C_R antagonist RS 102221 15 min prior to mCPP blocked the anxiogenic effect of mCPP on sociability in wildtype C57BL/6J mice (Figure 2), confirming that the mCPP-induced anxiogenic response in the social interaction test was mediated by 5-HT_2C_R [main effects of side (F_1,36 _= 64.29, p < 0.0001) and drug (F_3,36 _= 0.64, N.S.); side × drug interaction (F_3,36 _= 6.43, p = 0.001). Neither RS 102221 nor mCPP, administered alone or in combination, affected locomotor activity (data not shown).

#### Open field test

**Figure 3:**
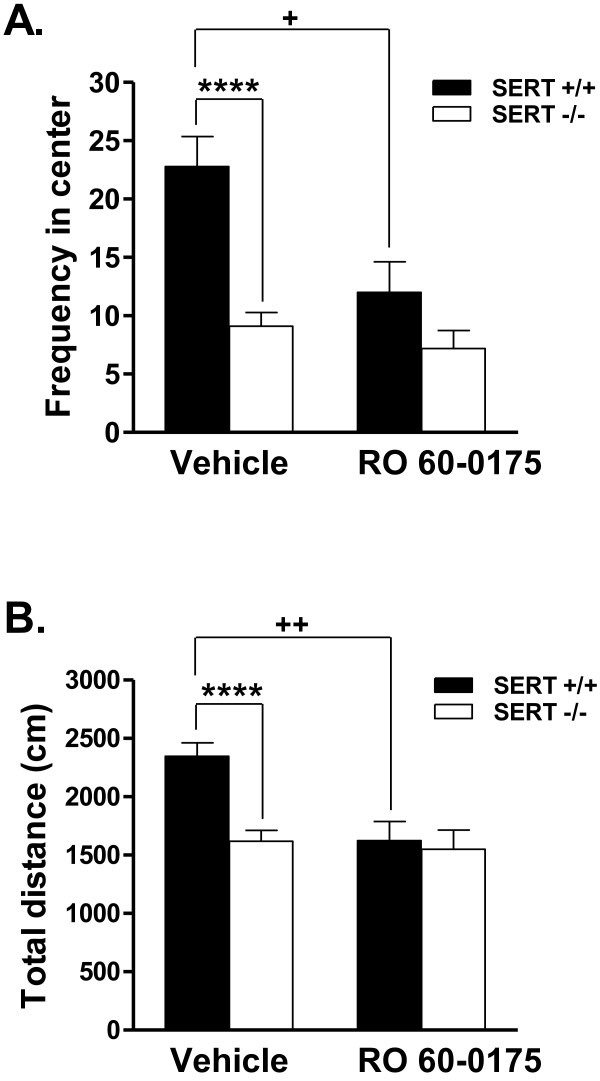
**Effects of RO 60-0175 in the open field test in SERT +/+ and -/- mice**. **3A.** Effects of RO 60-0175 on anxiety-like behavior. SERT +/+ and SERT -/- mice were given vehicle or RO 60-0175 4 mg/kg ip 30 min prior to testing. RO 60-0175 increased anxiety-like behavior (decreased visits to the center) in the open field in SERT +/+ mice, with no effect in SERT -/- mice. **3B.** Effects of RO 60-0175 on locomotor activity. RO 60-0175 4 mg/kg ip administered 30 min prior to testing decreased the total distance traveled in SERT +/+ mice compared to vehicle, with no effects in SERT -/- mice. Data represent the mean ± SEM, 9-10 animals per group. **** p < 0.0001 vs. SERT +/+ mice in the same drug condition; ++ p < 0.01 and + p < 0.05 vs. mice of the same genotype administered vehicle.

To further explore this apparent reduction in responsiveness of 5-HT_2C_R observed in the social interaction test, we tested the effects of the 5-HT_2C _agonist RO 60-0175 in the open field test. For the frequency in the center of the open field, there was a significant main effect of genotype (F_1,34 _= 20.70, p < 0.0001), a significant main effect of drug condition (F_1,34 _= 9.75, p = 0.004) and a significant genotype × drug condition interaction (F_1,34 _= 4.78, p = 0.036). Following vehicle, SERT -/- mice made fewer visits to the center of the open field compared to SERT +/+ mice (Figure 3A). In SERT +/+ mice, treatment with RO 60-0175 decreased the frequency of visits to the center of the open field to levels observed in SERT -/- mice, suggestive of an anxiogenic effect, whereas RO 60-0175 had no effect in SERT -/- mice (Figure 3A). Regarding the total distance traveled, there was a significant main effect of genotype (F_1,34 _= 8.23, p = 0.007), a significant main effect of drug condition (F_1,34 _= 8.05, p = 0.008) and a significant genotype × drug condition interaction (F_1,34 _= 5.50, p = 0.025). At baseline, SERT -/- mice displayed less locomotor activity than SERT +/+ mice (Figure 3B). There was also a reduction in locomotion in SERT +/+ mice after RO 60-0175 administration, to levels comparable to SERT -/- mice; RO 60-0175 did not alter locomotion in SERT -/- mice. Activity in periphery, however, remained unchanged (data not shown).

### RNA editing

**Figure 4:**
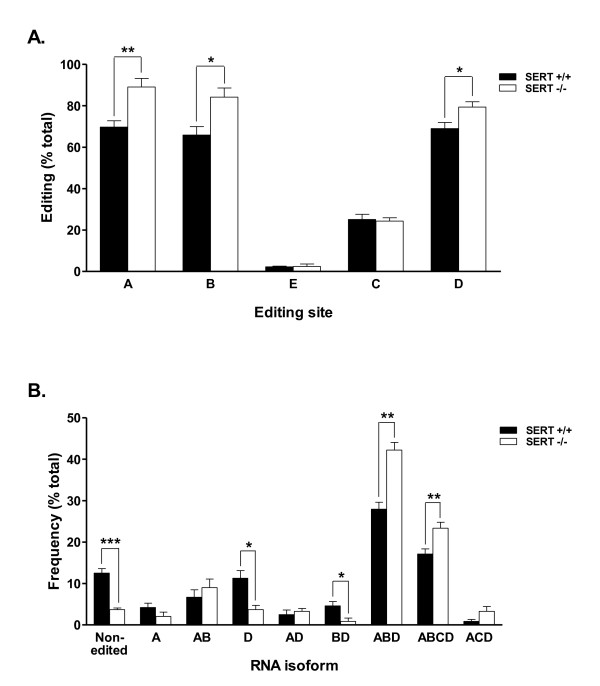
**RNA editing profiles of 5-HT_2C_R mRNA in amygdala of SERT +/+ and -/- mice**. **4A.** 5-HT_2C_R RNA editing per site. Compared to SERT +/+ mice, SERT -/- mice had significant increases in the editing rate of sites A, B and D, with no differences in editing rates for sites C or E. **4B.** Frequency of 5-HT_2C_R isoforms. Compared to SERT +/+ mice, SERT -/- mice evidenced a decrease in the expression of the non-edited, D and BD isoforms, and an increase in the ABD and ABCD isoforms. Data represent the mean ± SEM, 4 animals per group. *** p < 0.001, ** p < 0.01 and * p < 0.05 significantly different from SERT +/+ mice.

Positively sequenced clones were used to compare the 5-HT_2C_R RNA editing profiles of SERT +/+ and -/- mice. The change in the editing rate at each specific editing site is shown in Figure 4A. Compared to SERT +/+ mice, SERT -/- mice had significant increases in the editing rate of site A (89.1% vs. 69.7%, p = 0.009), site B (84.2% vs. 65.9%, p = 0.0227) and site D (79.3% vs. 68.9%, p = 0.04). No differences in editing rates between the two SERT genotypes were found for sites C or E. The frequency of the RNA isoforms expressed at least 3% in one of the genotypes is shown in Figure 4B. Compared to SERT +/+ mice, SERT -/- mice evidenced a significant decrease in the expression of the non-edited (3.7% vs. 12.5%, p = 0.0003), D (3.7% vs.11.3%, p = 0.0117) and BD (4.6% vs.1.1%, p = 0.0356) isoforms. Further, two of the 5-HT_2C_R RNA isoforms were significantly increased in SERT -/- mice compared to SERT +/+ mice; ABD (42.2% vs.27.9%, p = 0.0012) and ABCD (23.4% vs. 17.1%, p = 0.016). Overall, this comparison of 5-HT_2C_R RNA editing profiles shows an increase in editing in SERT -/- mice vs. SERT +/- mice which results in a shift from non/low editing isoforms toward highly/full edited isoforms.

## Discussion

To our knowledge, the present data document the first assessments of anxiety-related behavioral alterations elicited by 5-HT_2C_R agonists in SERT -/- mice. Specifically, in the social interaction test, there were no significant differences in baseline assessments (vehicle administration) between SERT +/+ and SERT -/- mice. However, the anxiogenic response induced by the 5-HT_2_R agonist mCPP in SERT +/+ mice was absent in SERT -/- mice. The role of 5-HT_2C_R in this anxiogenic response was confirmed by pretreatment with the selective 5-HT_2C_R antagonist RS 102221, which blocked the anxiogenic effect of mCPP in wildtype C57BL/6J mice. Others have previously shown that both systemic and local (intra-amygdala) administration of mCPP increases anxiety levels in rodents, indicating that these receptors - possibly in the basolateral amygdala - are responsible for the anxiogenic effect of mCPP [29,30].We also replicated previous findings from our lab showing that SERT -/- mice exhibit increased baseline anxiety-like behaviors in the open field test (for a review, see [1]). In addition, we showed that the anxiogenic response induced by the selective 5-HT_2C _agonist RO 60-0175 in the open field test in SERT +/+ mice is abolished in SERT -/- mice.

Using autoradiography to determine binding sites and *in situ *hybridization for mRNA content, previous reports from our lab indicate that 5-HT_2C_R mRNA levels are unaltered in amygdala of SERT -/- mice, whereas 5-HT_2C_R binding sites are significantly increased in this region [15]. We therefore hypothesized that differences in RNA editing levels might account for this apparent discrepancy between levels of binding sites and the 5-HT_2C_R responsiveness to agonist stimulation. Given previous reports indicating that intra-amygdala injections of mCPP were able to elicit anxiogenic responses in rodents [29,30], and the known role of this brain region in rodent and human anxiety, we focused our efforts on characterizing the RNA editing profile of 5-HT_2C_R in amygdala of SERT -/- mice compared to that of their SERT +/+ littermates.

SERT -/- mice had significantly decreased frequencies of non-edited, D and BD isoforms, as well as a significantly increased frequencies of the ABCD and ABD isoforms, the latter being the major isoform present in both SERT +/+ and -/- mice. ABCD codes for the VSV variant of the 5-HT_2C_R, and has been shown to exhibit reductions in receptor signaling both in agonist-elicited and intrinsic activity [16,31]. The major isoform ABD codes for the variant VNV together with the AD isoform, which was slightly increased in SERT -/- mice compared to SERT +/+ mice. Previous studies also show that the VNV is the major 5-HT_2C_R isoform present in C57BL/6J mice [32,33]. The VNV variant has reduced basal activity with no alteration in the potency of 5-HT stimulation [16,31]. Thus, the present results suggest that SERT gene deletion shifts the RNA editing profile of the 5-HT_2C_R pre-mRNA population toward more edited, less active isoforms. This might explain the lack of effect of 5-HT_2C _agonists in both the social interaction and open field tests in SERT -/- mice at doses which were anxiogenic in SERT +/+ mice. It is important, however, to emphasize that the aforementioned reports indicating pharmacological differences among 5-HT_2C_R RNA editing isoforms were conducted with human and rat clones of each RNA isoform heterologously expressed, therefore caution is required when comparing the values from the present *in vivo *study conducted in mice [16,31].

Previous reports have shown that pharmacological manipulations of serotonergic tone have an impact on 5-HT_2C_R RNA editing, either by direct 5-HT_2C_R activation by the non-selective 5-HT_2 _agonist DOI or by chronic fluoxetine (a selective serotonin reuptake inhibitor (SSRI)) treatment. Gurevich and colleagues [21] found that 129Sv mice treated with chronic fluoxetine exhibit significantly increased editing in site D and significantly decreased editing in site E. Chronic fluoxetine treatment in C57BL/6J mice, however, led to modest, non-significant changes in 5-HT_2C_R RNA editing, whereas the same treatment in BALB/c mice led to significant increases in editing of sites A, B, C and D [22]. In SERT -/- mice, extracellular 5-HT levels are increased 3-6 fold in brain [1,34]. Our current results suggest that, as a result of a targeted gene deletion of SERT, the increased extracellular levels of 5-HT alters 5-HT_2C_R RNA editing. In addition, these results suggest that targeted SERT gene deletion has a more profound impact than 28 days of treatment with fluoxetine in C57BL/6J wildtype mice [21]. However, it is important to note that the prior studies analyzed 5-HT_2C_R RNA editing in forebrain neocortex [21,22,35], whereas the current study analyzed 5-HT_2C_R RNA editing in amygdala; thus, a direct comparison of these studies with the current studies is limited by the anatomical differences.

The observed increase in the frequency of 5-HT_2C_R RNA editing in SERT -/- mice might also explain the apparent paradoxical upregulation of the number of 5-HT_2C_R binding sites previously observed in SERT -/- mice [15]. It has been shown that RNA editing also alters the ratio of alternative splicing variants, promoting the generation of the full mRNA variant coding for the functional protein *in vitro *[36], and recently *in vivo *[23]. Thus, the increased RNA editing observed here in SERT -/- mice might be related to the previously observed increase in surface expression of 5-HT_2C_R [15]. Another plausible link between RNA editing and 5-HT_2C_R upregulation is receptor desensitization [5]. It is known that the non-edited 5-HT_2C_R isoform exhibits the highest constitutive activity and is present mainly intracellularly, whereas more edited isoforms are present largely as membrane-bound receptors and are more resistant to desensitization, at least *in vitro *[37,38]. The observed increase in 5-HT_2C_R RNA editing in SERT -/- mice, which generates receptor isoforms with less efficacious signaling and reduced basal activity, is in line with previous findings of a reduction in DOI-induced arachidonic acid release in several brain regions, including the basolateral amygdaloid complex, in SERT -/- mice [13,14]. However, the concomitant activation of 5-HT_2A _receptors does not allow a claim to be made for reduced activity of 5-HT_2C_R in those studies, especially as other signaling pathways for 5HT_2C_R receptors exist (e.g., PLC/IP_3_).

There is considerable evidence suggesting the involvement of 5-HT_2C_R in anxiety-related behaviors, although there is still debate about the precise role of this receptor in anxiety (for a review, see [2]). For example, it has been shown that activation of 5-HT_2C_R mediates the anxiogenic-like effects elicited by the non-selective 5-HT_2C _agonist mCPP in rodents, replicated in the current studies, and in humans [2,39,40]. Similarly, selective 5-HT_2C_R antagonists have been shown to exert anxiolytic effects in several animal models of anxiety in some reports [41,42], but not in others [43,44]. The current results are in line with reports of initial anxiogenic-like effects of SSRIs treatment in both humans and in several animal models of anxiety [45]. The current data also show that, as indicated above, varying SERT expression can have profound consequences on the functional status of postsynaptic 5-HT_2C_R receptors, as expected from the marked increases in extracellular levels of 5-HT found in SERT -/- mice [34]. These results also suggest that polymorphisms affecting SERT expression might exert a modulatory effect on the functional status of 5-HT_2C_R in humans. This might have implications for personalized medicine, as several selective 5-HT_2C_R agonists are being proposed as anti-obesity agents that have now advanced to clinical trials [46], in addition to the reported potential use of 5-HT_2C_R antagonists as anxiolytics [41].

The current studies focused on the analysis of RNA editing in amygdala, a key region involved in fear and anxiety. However, the circuit controlling anxiety-related traits and responses spans multiple regions. It will be of interest for future research to examine different brain areas to evaluate potential region-specific alterations in 5-HT_2C_R RNA editing frequencies, based on previous studies showing brain region-specific alterations in tissue 5-HT content, and in 5-HT synthesis and turnover rates [9,47,48]. A detailed characterization of the role of 5-HT_2C_R in amygdala control and in alterations in its RNA editing profile might also require a finer dissection (such as laser-caption microdissection) of the different subregions of the heterogeneous amygdala structure.

## Conclusions

In summary, for the first time, we report functional alterations of 5-HT_2C_R-mediated responses to agonist stimulation in SERT -/- mice, as observed in the social interaction and open field paradigms. Further, we suggest that this alteration could be, at least in part, be explained by the significant increases in RNA editing of 5-HT_2C_R in the amygdala of SERT -/- mice that generates less active receptor isoforms. These findings will help to unravel the role of 5-HT neurotransmission in amygdala activity, especially in terms of alterations in SERT expression reported in humans with different alleles for the SERT promoter (5-HTTLPR s and l alleles) and other polymorphisms affecting SERT expression that have been found to be relevant in neuropsychiatric disorders [49-51]. Additional efforts are needed to further dissect the role of the 5-HT_2C_R among different amygdala subnuclei and in different neuronal types, to further understand the physiological relevance of 5-HT_2C_R editing in this and other brain regions, in addition to the role of 5-HT_2C_R in neuropsychiatric disorders.

## Methods

### Animals

Male SERT +/+ and -/- mice were originally produced by homologous recombination in ES cells as previously described [52], and are currently the product of ~20-24 heterozygous backcrosses with wildtype mice on a C57BL/6J genetic background. Commercial wildtype C57BL/6J mice (Jackson Laboratory, Bar Harbor, ME) were used for the antagonism experiment. The animals weighed ~20-35 g at the time of the experiments, and were housed in groups of 3-5 per cage with food and water available *ad libitum*. The animals were maintained on a 12-h light/dark cycle (lights on 0600 hours) in a facility approved by the American Association for Accreditation of Laboratory Animal Care. All experiments adhered to the guidelines of the National Institutes of Health, and were approved by the National Institute of Mental Health Animal Care and Use Committee.

### Drugs and drug administration

The following compounds were used: (i) the 5-HT_2B/2C _agonist 1-(3-chlorophenly)piperazine (mCPP) (Tocris Bioscience, Ellisville, MO), (ii) the selective 5-HT_2C _agonist (αS)-6-Chloro-5-fluoro-α-methyl-1H-indole-1-ethanamine fumarate (RO 60-0175), and (iii) the selective 5-HT_2C _antagonist 8-[5-(2,4-Dimethoxy-5-(4-trifluoromethylphenylsulphonamido)phenyl-5-oxopentyl]-1,3,8-triazaspiro[4.5]decane-2,4-dione hydrochloride (RS 102221) (Tocris Bioscience, Ellisville, MO). mCPP was administered at a dose of 1 mg/kg and RO 60-0175 was administered at a dose of 4 mg/kg, based on previous investigations that showed behavioral effects at these doses [29,43] and preliminary pilot studies performed in our lab. RS 102221 was administered at 1 mg/kg based on previous investigations [53]. mCPP and RO 60-0175 were dissolved in saline (sterile 0.9% NaCl solution), and RS 10221 was dissolved in 1% DMSO and saline. Drugs were injected via intraperitoneal (ip) injection (injection volume 10 ml/kg) 30 min prior to behavioral testing. In the antagonism study, RS 102221 was injected 15 min prior to mCPP.

### Behavioral paradigms

A separate cohort of animals was used for each behavioral study. On test days, animals were moved in their home cage to a dimly lit testing room 1 h prior to experiments. All behavioral experiments were carried out between 1000 and 1400 hours.

#### Social interaction test

The social interaction test was used because it can detect the anxiolytic and anxiogenic effects of serotonergic agents [54,55]. SERT +/+ and -/- mice were injected with either vehicle (saline) or mCPP. Thirty min later, mice were tested in an automated three-chamber box as described previously [56]. Dividing walls had retractable doorways allowing access into each chamber. The automated box had photocells embedded in each doorway to allow quantification of the number of entries and the duration in each chamber of the social test box. The chambers of the apparatus were cleaned with water and dried with paper towels between each trial. At the end of each test day, the apparatus was sprayed with 70% ethanol and wiped clean with paper towels. The test has three 10-min phases: (1) Center habituation - the test mouse was first placed in the middle chamber and allowed to explore, with the doorways into the two side chambers closed; (2) Side chamber habituation - the mouse was allowed to explore the entire social test box, with the doorways into the two side chambers open, and (3) Sociability - after the second habituation period, the test mouse was enclosed in the center compartment of the social test box, and an unfamiliar mouse ("stranger," an adult C57BL/6J male) was enclosed in a wire cage (11 cm height, 10.5 bottom diameter, bars spaced 1 cm apart; Galaxy Cup; Spectrum Diversified Designs, Inc., Streetsboro, OH) and placed in a side chamber, and a similar empty wire cage was placed in the other side chamber. The location of the stranger alternated between the left and the right sides of the social test box between subjects. Following placement of the stranger mouse, the doors were reopened, and the subject was allowed to explore the entire social test box. The automated testing system recorded the amount of time spent and the number of entries in each chamber. In addition, the time spent sniffing each wire cage was recorded by an experimenter blind to the administered drug.

#### Open field test

As pilot studies indicated that a range of doses of mCPP (0 - 5 mg/kg) did not elicit anxiogenic effects in the open field test, we evaluated the effects of RO 60-0175, a selective 5-HT_2C_R agonist. SERT +/+ and -/- mice were injected with either vehicle or RO 60-0175. Thirty min later, mice were placed in the corner of a novel open field arena (40 × 40 × 35) and were allowed to explore for 5 min. Behaviors, including distance traveled (cm) and frequency of visits to center (20 × 20 cm), were recorded using the Noldus Ethovision Video Tracking system (Noldus Information Technology, Leesburg, VA).

### RNA editing

Determinations of RNA editing profiles were performed in a separate cohort of SERT +/+ and -/- mice. Amygdala samples were obtained as previously described [32]. Mice were sacrificed and brains were rapidly removed and placed in a brain block matrix. 1 mm coronal sections encompassing the amygdala region were dissected (posterior to the optic chiasm and anterior to the pons as ventral surface landmarks). From coronal sections, tissue containing visible amygdala nuclei was dissected using the *rhinal sulcus *as a guide. The tissues from both hemispheres were collected together.

Total RNA was extracted using miRvana PARIS Kit (Ambion, Austin, TX). 480 ng were used in first-strand cDNA synthesis using SuperScript III First-Strand SuperMix (Invitrogen, Carlsbad, CA) using the gene-specific primer CGGCGTAGGACGTAGATCGTTAAG [33]. Amplification of the edited region was performed using primers sense (5'-TGTGCTATTTTCAACTGCGTCCATCATG), antisense (5'-CGGCGTAGGACGTAGATCGTTAAG) and Master Mix (Promega, Madison, WI). PCR products were cloned into pCR2.1 vector (Invitrogen, Carlsbad, CA) and used for transformation in *E. coli*. From each animal, isolated colonies were randomly chosen for plasmid DNA isolation (Qiagen, Valencia, CA) and bidirectionally sequenced with M13 primers at the National Institute of Neurological Disorders and Stroke (NINDS) intramural DNA sequencing core facility. Raw chromatograms from 60 positively sequenced colonies per animal (240 per genotype) were analyzed for changes in the editing region previously described.

### Statistical analysis

For each experiment, data were analyzed using two-way (genotype × drug condition) or three-way (genotype × drug condition × side) analyses of variance (ANOVAs), or by t-tests when only two groups were compared. Post-hoc comparisons between genotypes or between drug conditions were conducted using t-tests. Significance was based on p < 0.05.

## Authors' contributions

PRM conceived, designed and supervised the experiments, analyzed and interpreted the data, and wrote the manuscript. MAF participated in the design of studies, performed the pilot studies, performed the statistical analysis for the behavioral section, and contributed significantly to the writing of the manuscript. CLJ carried out the cloning experiments. JLL carried out the social interaction test experiments. HTF carried out the open field test experiments. JRW participated in the design and execution of the molecular experiments and helped with the RNA editing sequence analysis. DLM supervised PRM's work, participated in the design and coordination of the experiments, and helped to write the manuscript. All authors read the manuscript, provided critical input, and approved the final manuscript.

## References

[B1] Murphy DL Lesch KP Targeting the murine serotonin transporter: insights into human neurobiology Nat Rev Neurosci 2008 9 2 85 96 10.1038/nrn2284 18209729

[B2] Millan MJ The neurobiology and control of anxious states Prog Neurobiol 2003 70 2 83 244 10.1016/S0301-0082(03)00087-X 12927745

[B3] Fink KB Gothert M 5-HT receptor regulation of neurotransmitter release Pharmacol Rev 2007 59 4 360 417 18160701 10.1124/pr.107.07103

[B4] Heisler LK Pronchuk N Nonogaki K Zhou L Raber J Tung L Yeo GS O'Rahilly S Colmers WF Elmquist JK Serotonin activates the hypothalamic-pituitary-adrenal axis via serotonin 2C receptor stimulation J Neurosci 2007 27 26 6956 6964 10.1523/JNEUROSCI.2584-06.2007 17596444 PMC6672238

[B5] Werry TD Loiacono R Sexton PM Christopoulos A RNA editing of the serotonin 5HT2C receptor and its effects on cell signalling, pharmacology and brain function Pharmacol Ther 2008 119 1 7 23 10.1016/j.pharmthera.2008.03.012 18554725

[B6] Berg KA Maayani S Goldfarb J Scaramellini C Leff P Clarke WP Effector pathway-dependent relative efficacy at serotonin type 2A and 2C receptors: evidence for agonist-directed trafficking of receptor stimulus Mol Pharmacol 1998 54 1 94 104 9658194

[B7] McGrew L Chang MS Sanders-Bush E Phospholipase D activation by endogenous 5-hydroxytryptamine 2C receptors is mediated by Galpha13 and pertussis toxin-insensitive Gbetagamma subunits Mol Pharmacol 2002 62 6 1339 1343 10.1124/mol.62.6.1339 12435801

[B8] Werry TD Gregory KJ Sexton PM Christopoulos A Characterization of serotonin 5-HT2C receptor signaling to extracellular signal-regulated kinases 1 and 2 J Neurochem 2005 93 6 1603 1615 10.1111/j.1471-4159.2005.03161.x 15935077

[B9] Fox MA Jensen CL French HT Stein AR Huang SJ Tolliver TJ Murphy DL Neurochemical, behavioral, and physiological effects of pharmacologically enhanced serotonin levels in serotonin transporter (SERT)-deficient mice Psychopharmacology (Berl) 2008 201 2 203 218 10.1007/s00213-008-1268-7 18712364 PMC2584159

[B10] Heisler LK Chu HM Tecott LH Epilepsy and obesity in serotonin 5-HT2C receptor mutant mice Ann N Y Acad Sci 1998 861 74 78 10.1111/j.1749-6632.1998.tb10175.x 9928241

[B11] Nonogaki K Strack AM Dallman MF Tecott LH Leptin-independent hyperphagia and type 2 diabetes in mice with a mutated serotonin 5-HT2C receptor gene Nat Med 1998 4 10 1152 1156 10.1038/2647 9771748

[B12] Miller KJ Serotonin 5-ht2c receptor agonists: potential for the treatment of obesity Mol Interv 2005 5 5 282 291 10.1124/mi.5.5.8 16249524

[B13] Qu Y Villacreses N Murphy DL Rapoport SI 5-HT2A/2C receptor signaling via phospholipase A2 and arachidonic acid is attenuated in mice lacking the serotonin reuptake transporter Psychopharmacology (Berl) 2005 180 1 12 20 10.1007/s00213-005-2231-5 15834538

[B14] Basselin M Fox MA Chang L Bell JM Greenstein D Chen M Murphy DL Rapoport SI Imaging elevated brain arachidonic acid signaling in unanesthetized serotonin transporter (5-HTT)-deficient mice Neuropsychopharmacology 2009 34 7 1695 1709 10.1038/npp.2008.227 19145225 PMC2700347

[B15] Li Q Wichems CH Ma L Van de Kar LD Garcia F Murphy DL Brain region-specific alterations of 5-HT2A and 5-HT2C receptors in serotonin transporter knockout mice J Neurochem 2003 84 6 1256 1265 10.1046/j.1471-4159.2003.01607.x 12614326

[B16] Burns CM Chu H Rueter SM Hutchinson LK Canton H Sanders-Bush E Emeson RB Regulation of serotonin-2C receptor G-protein coupling by RNA editing Nature 1997 387 6630 303 308 10.1038/387303a0 9153397

[B17] Niswender CM Copeland SC Herrick-Davis K Emeson RB Sanders-Bush E RNA editing of the human serotonin 5-hydroxytryptamine 2C receptor silences constitutive activity J Biol Chem 1999 274 14 9472 9478 10.1074/jbc.274.14.9472 10092629

[B18] Wang Q O'Brien PJ Chen CX Cho DS Murray JM Nishikura K Altered G protein-coupling functions of RNA editing isoform and splicing variant serotonin2C receptors J Neurochem 2000 74 3 1290 1300 10.1046/j.1471-4159.2000.741290.x 10693963

[B19] Price RD Sanders-Bush E RNA editing of the human serotonin 5-HT(2C) receptor delays agonist-stimulated calcium release Mol Pharmacol 2000 58 4 859 862 10999958 10.1124/mol.58.4.859

[B20] Berg KA Clarke WP Cunningham KA Spampinato U Fine-tuning serotonin2c receptor function in the brain: molecular and functional implications Neuropharmacology 2008 55 6 969 976 10.1016/j.neuropharm.2008.06.014 18602407 PMC3124806

[B21] Gurevich I Tamir H Arango V Dwork AJ Mann JJ Schmauss C Altered editing of serotonin 2C receptor pre-mRNA in the prefrontal cortex of depressed suicide victims Neuron 2002 34 3 349 356 10.1016/S0896-6273(02)00660-8 11988167

[B22] Englander MT Dulawa SC Bhansali P Schmauss C How stress and fluoxetine modulate serotonin 2C receptor pre-mRNA editing J Neurosci 2005 25 3 648 651 10.1523/JNEUROSCI.3895-04.2005 15659601 PMC6725319

[B23] Dracheva S Chin B Haroutunian V Altered serotonin 2C receptor RNA splicing in suicide: association with editing Neuroreport 2008 19 3 379 382 10.1097/WNR.0b013e3282f556d2 18303585

[B24] Gardiner K Du Y A-to-I editing of the 5HT2C receptor and behaviour Brief Funct Genomic Proteomic 2006 5 1 37 42 10.1093/bfgp/ell006 16769676

[B25] Bhansali P Dunning J Singer SE David L Schmauss C Early life stress alters adult serotonin 2C receptor pre-mRNA editing and expression of the alpha subunit of the heterotrimeric G-protein G q J Neurosci 2007 27 6 1467 1473 10.1523/JNEUROSCI.4632-06.2007 17287521 PMC6673584

[B26] Oler JA Fox AS Shelton SE Christian BT Murali D Oakes TR Davidson RJ Kalin NH Serotonin transporter availability in the amygdala and bed nucleus of the stria terminalis predicts anxious temperament and brain glucose metabolic activity J Neurosci 2009 29 32 9961 9966 10.1523/JNEUROSCI.0795-09.2009 19675230 PMC2756094

[B27] Hariri AR Drabant EM Munoz KE Kolachana BS Mattay VS Egan MF Weinberger DR A susceptibility gene for affective disorders and the response of the human amygdala Arch Gen Psychiatry 2005 62 2 146 152 10.1001/archpsyc.62.2.146 15699291

[B28] Hariri AR Mattay VS Tessitore A Kolachana B Fera F Goldman D Egan MF Weinberger DR Serotonin transporter genetic variation and the response of the human amygdala Science 2002 297 5580 400 403 10.1126/science.1071829 12130784

[B29] Bagdy G Graf M Anheuer ZE Modos EA Kantor S Anxiety-like effects induced by acute fluoxetine, sertraline or m-CPP treatment are reversed by pretreatment with the 5-HT2C receptor antagonist SB-242084 but not the 5-HT1A receptor antagonist WAY-100635 Int J Neuropsychopharmacol 2001 4 4 399 408 10.1017/S1461145701002632 11806866

[B30] Campbell BM Merchant KM Serotonin 2C receptors within the basolateral amygdala induce acute fear-like responses in an open-field environment Brain Res 2003 993 1-2 1 9 10.1016/S0006-8993(03)03384-5 14642825

[B31] Herrick-Davis K Grinde E Niswender CM Serotonin 5-HT2C receptor RNA editing alters receptor basal activity: implications for serotonergic signal transduction J Neurochem 1999 73 4 1711 1717 10.1046/j.1471-4159.1999.731711.x 10501219

[B32] Hackler EA Airey DC Shannon CC Sodhi MS Sanders-Bush E 5-HT(2C) receptor RNA editing in the amygdala of C57BL/6J, DBA/2J, and BALB/cJ mice Neurosci Res 2006 55 1 96 104 10.1016/j.neures.2006.02.005 16580757

[B33] Du Y Davisson MT Kafadar K Gardiner K A-to-I pre-mRNA editing of the serotonin 2C receptor: comparisons among inbred mouse strains Gene 2006 382 39 46 10.1016/j.gene.2006.06.007 16904273

[B34] Mathews TA Fedele DE Coppelli FM Avila AM Murphy DL Andrews AM Gene dose-dependent alterations in extraneuronal serotonin but not dopamine in mice with reduced serotonin transporter expression J Neurosci Methods 2004 140 1-2 169 181 10.1016/j.jneumeth.2004.05.017 15589347

[B35] Gurevich I Englander MT Adlersberg M Siegal NB Schmauss C Modulation of serotonin 2C receptor editing by sustained changes in serotonergic neurotransmission J Neurosci 2002 22 24 10529 10532 12486144 10.1523/JNEUROSCI.22-24-10529.2002 PMC6758441

[B36] Flomen R Knight J Sham P Kerwin R Makoff A Evidence that RNA editing modulates splice site selection in the 5-HT2C receptor gene Nucleic Acids Res 2004 32 7 2113 2122 10.1093/nar/gkh536 15087490 PMC407821

[B37] Marion S Weiner DM Caron MG RNA editing induces variation in desensitization and trafficking of 5-hydroxytryptamine 2c receptor isoforms J Biol Chem 2004 279 4 2945 2954 10.1074/jbc.M308742200 14602721

[B38] Porter RH Malcolm CS Allen NH Lamb H Revell DF Sheardown MJ Agonist-induced functional desensitization of recombinant human 5-HT2 receptors expressed in CHO-K1 cells Biochem Pharmacol 2001 62 4 431 438 10.1016/S0006-2952(01)00677-3 11448452

[B39] Silverstone PH Rue JE Franklin M Hallis K Camplin G Laver D Cowen PJ The effects of administration of mCPP on psychological, cognitive, cardiovascular, hormonal and MHPG measurements in human volunteers Int Clin Psychopharmacol 1994 9 3 173 178 10.1097/00004850-199409000-00005 7814826

[B40] Murphy DL Mueller EA Hill JL Tolliver TJ Jacobsen FM Comparative anxiogenic, neuroendocrine, and other physiologic effects of m-chlorophenylpiperazine given intravenously or orally to healthy volunteers Psychopharmacology (Berl) 1989 98 2 275 282 10.1007/BF00444705 2502799

[B41] Kennett GA Wood MD Bright F Trail B Riley G Holland V Avenell KY Stean T Upton N Bromidge S SB 242084, a selective and brain penetrant 5-HT2C receptor antagonist Neuropharmacology 1997 36 4-5 609 620 10.1016/S0028-3908(97)00038-5 9225286

[B42] Martin JR Ballard TM Higgins GA Influence of the 5-HT2C receptor antagonist, SB-242084, in tests of anxiety Pharmacol Biochem Behav 2002 71 4 615 625 10.1016/S0091-3057(01)00713-4 11888553

[B43] Nic Dhonnchadha BA Bourin M Hascoet M Anxiolytic-like effects of 5-HT2 ligands on three mouse models of anxiety Behav Brain Res 2003 140 1-2 203 214 10.1016/S0166-4328(02)00311-X 12644293

[B44] Jenck F Moreau JL Berendsen HH Boes M Broekkamp CL Martin JR Wichmann J Van Delft AM Antiaversive effects of 5HT2C receptor agonists and fluoxetine in a model of panic-like anxiety in rats Eur Neuropsychopharmacol 1998 8 3 161 168 10.1016/S0924-977X(97)00055-2 9716307

[B45] Salchner P Singewald N 5-HT receptor subtypes involved in the anxiogenic-like action and associated Fos response of acute fluoxetine treatment in rats Psychopharmacology (Berl) 2006 185 3 282 288 10.1007/s00213-005-0247-5 16521035

[B46] Dutton AC Barnes NM Anti-obesity pharmacotherapy: Future perspectives utilising 5-HT2C receptor agonists Drug Discovery Today: Therapeutic Strategies 2006 3 4 577 583 10.1016/j.ddstr.2006.11.005

[B47] Kim DK Tolliver TJ Huang SJ Martin BJ Andrews AM Wichems C Holmes A Lesch KP Murphy DL Altered serotonin synthesis, turnover and dynamic regulation in multiple brain regions of mice lacking the serotonin transporter Neuropharmacology 2005 49 6 798 810 10.1016/j.neuropharm.2005.08.010 16183083

[B48] Ren-Patterson RF Cochran LW Holmes A Lesch KP Lu B Murphy DL Gender-dependent modulation of brain monoamines and anxiety-like behaviors in mice with genetic serotonin transporter and BDNF deficiencies Cell Mol Neurobiol 2006 26 4-6 755 780 10.1007/s10571-006-9048-6 17029036 PMC11520647

[B49] Wendland JR Moya PR Kruse MR Ren-Patterson RF Jensen CL Timpano KR Murphy DL A novel, putative gain-of-function haplotype at SLC6A4 associates with obsessive-compulsive disorder Hum Mol Genet 2008 17 5 717 723 10.1093/hmg/ddm343 18055562

[B50] Hu XZ Lipsky RH Zhu G Akhtar LA Taubman J Greenberg BD Xu K Arnold PD Richter MA Kennedy JL Serotonin transporter promoter gain-of-function genotypes are linked to obsessive-compulsive disorder Am J Hum Genet 2006 78 5 815 826 10.1086/503850 16642437 PMC1474042

[B51] Murphy DL Lerner A Rudnick G Lesch KP Serotonin transporter: gene, genetic disorders, and pharmacogenetics Mol Interv 2004 4 2 109 123 10.1124/mi.4.2.8 15087484

[B52] Bengel D Murphy DL Andrews AM Wichems CH Feltner D Heils A Mossner R Westphal H Lesch KP Altered brain serotonin homeostasis and locomotor insensitivity to 3, 4-methylenedioxymethamphetamine ("Ecstasy") in serotonin transporter-deficient mice Mol Pharmacol 1998 53 4 649 655 9547354 10.1124/mol.53.4.649

[B53] Kuznetsova EG Amstislavskaya TG Shefer EA Popova NK Effect of 5-HT2C receptor antagonist RS 102221 on mouse behavior Bull Exp Biol Med 2006 142 1 76 79 10.1007/s10517-006-0296-8 17369908

[B54] Gonzalez LE Andrews N File SE 5-HT1A and benzodiazepine receptors in the basolateral amygdala modulate anxiety in the social interaction test, but not in the elevated plus-maze Brain Res 1996 732 1-2 145 153 10.1016/0006-8993(96)00517-3 8891278

[B55] Overstreet DH Knapp DJ Moy SS Breese GR A 5-HT1A agonist and a 5-HT2c antagonist reduce social interaction deficit induced by multiple ethanol withdrawals in rats Psychopharmacology (Berl) 2003 167 4 344 352 12677355 10.1007/s00213-003-1425-y PMC2865243

[B56] Nadler JJ Moy SS Dold G Trang D Simmons N Perez A Young NB Barbaro RP Piven J Magnuson TR Automated apparatus for quantitation of social approach behaviors in mice Genes Brain Behav 2004 3 5 303 314 10.1111/j.1601-183X.2004.00071.x 15344923

